# Fatty acid metabolism-related genes are associated with flavor-presenting aldehydes in Chinese local chicken

**DOI:** 10.3389/fgene.2022.902180

**Published:** 2022-08-12

**Authors:** Xiaoya Yuan, Huanxian Cui, Yuxi Jin, Wenjuan Zhao, Xiaojing Liu, Yongli Wang, Jiqiang Ding, Li Liu, Jie Wen, Guiping Zhao

**Affiliations:** State Key Laboratory of Animal Nutrition, Institute of Animal Sciences, Chinese Academy of Agricultural Sciences, Beijing, China

**Keywords:** aldehydes, genome-wide association study, chicken flavor, lipid metabolism, fatty acid, phospholipid

## Abstract

Aldehydes are primary volatile organic compounds (VOCs) in local Chinese chicken meat and contribute green grass, fatty, citrus, and bitter almond aromas to chicken meat. To understand the genetic basis of these aldehyde VOC aromas, we used approximately 500 Chinese Jingxing Yellow (JXY) chickens to conduct genome-wide association studies (GWAS) on the flavor traits with the data of single nucleotide polymorphisms (SNPs) and insertions and deletions (INDELs). In total, 501 association variants (253 SNPs and 248 INDELs) were found to be suggestively (SNPs: *p-*value *<* 2.77e-06 and INDELs: *p-*value *<* 3.78e-05) associated with total aldehydes (the sum of nine aldehydes), hexanal, heptanal, benzaldehyde, (E,E)-2,4-nonadienal, octanal, (E)-2-decenal, nonanal, decanal, and octadecanal. Of them, six SNPs and 23 INDELs reached a genome-wide significance level (SNPs: *p-*value *<* 1.38e-07 and INDELs: *p-*value *<* 1.89e-06). Potential candidate aldehyde genes were functionally annotated for lipid metabolism, especially fatty acid-related pathways and phospholipid-related gene ontology (GO) terms. Moreover, the GWAS analysis of total aldehydes, hexanal, and nonanal generated the most significant signals, and phenotypic content differed between different genotypes at candidate gene-related loci. For total aldehydes and hexanal traits, candidate genes were annotated based on the significant and suggestive variants on chromosomes 3 and 8 with highly polymorphic linkage blocks. The following candidate genes were also identified: *GALM*, *MAP4K3*, *GPCPD1*, *RPS6KA2, CRLS1*, *ASAP1*, *TRMT6*, *SDC1*, *PUM2*, *ALDH9A1*, *MGST3*, *GMEB1*, *MECR*, *LDLRAP1*, *GPAM* and *ACSL5*. We also found that polyunsaturated fatty acids (PUFAs) (C18:2n6c linoleic acid and C18:3n3 linolenic acid) were significantly correlated with total aldehydes and hexanal contents. PUFAs are important aldehyde precursors, and consistently, our results suggested that candidate genes involved in fatty acid pathways and phospholipid GO terms were identified in association loci. This work provides an understanding of the genetic basis of aldehyde formation, which is a key flavor-forming compound.

## Introduction

Chicken is an indispensable meat product in most daily life, and chicken consumption has been steadily increasing over recent years (Petracci and Cavani, 2012; Park et al., 2020). Chicken meat is considered a healthy diet choice because it is high in protein, low in fat and cholesterol, and easier to handle in terms of portions (De Liu et al., 2012; Katemala et al., 2021). However, a rapid upscaling in meat productivity, under high-intensity genetic selection, could be a major factor contributing to a decline in meat quality and flavor. To improve chicken flavor, while increasing growth rates in breeding programs, flavor variability sources in chicken meat must be identified to understand the genetic architecture and define cost-effective selection methods.

Flavor is a complex multifactorial trait involving combinations of taste, mouth-feel, and aroma perception. In chicken meat, flavor is thermally derived, with the Maillard reaction, and the thermal degradation of lipids, and combinations thereof, mainly responsible for flavor and aroma generation (Jayasena et al., 2013). Moreover, as flavor phenotyping is expensive for high-throughput assays and subject to environmental variation, most breeders do not focus on flavor quality (Tieman et al., 2017). Aldehydes are key aroma compounds in chicken meat, soup, and liver, and primarily include hexanal, benzaldehyde, nonanal, (E,E)-2,4-nonadienal, and (E)-2-decenal, and are mainly formed from lipid oxidation and decomposition (Fan et al., 2018; Feng et al., 2018; Chen et al., 2020). Our previous study showed that aldehydes, which accounted for 72.92% of the total aroma volatile organic compounds (VOCs) in JXY chickens, were the most abundant aroma VOCs in nearly 1000 local Chinese chicken meat samples, with hexanal being the highest VOC (Jin et al., 2021). Therefore, our previous research laid the foundations for identifying and characterizing molecular variants in major aldehydes.

Molecular markers are tools used to detect genetic associations and predict phenotypes. The genome-wide association study (GWAS) approach has been successfully used to investigate significant single nucleotide polymorphisms (SNPs) of VOCs in plants and fruit. The genetic loci of target flavor chemicals in tomatoes were identified by GWAS, and included sugars, acids, and volatiles (Tieman et al., 2017). The genetic architecture of VOCs in blueberry flavor was elucidated using metabolite GWAS, and fatty acid-derived VOC related genes were identified (Ferrão et al., 2020). Moreover, a quality trait locus (QTL) study of 17 sensory attributes (including appearance, taste, flavor, and texture) identified the genetic factors of pig influence on the perception of sensory attributes generated during dry-cured ham processing (Pena et al., 2013). However, variants and genes associated with VOCs in chicken meat remain unknown.

The Chinese local chicken breed is known for its excellent and unique taste and flavor. In this study, we performed GWAS on aldehyde flavor VOCs in local high-quality yellow chickens and identified candidate genes potentially impacting the formation and metabolism pathways of chicken aldehyde compounds. These candidate genes contribute to an improved understanding of the genetic basis underpinning the main VOC traits, and they provide breeding tools to improve flavors in chickens.

## Material and methods

### Animals and sample collection

The animals used in this study were JXY female chickens provided by the Chinese Academy of Agricultural Sciences (CAAS) (Beijing, China). Two JXY chicken lines originated from the same base population of Jingxing 100, whose main selection trait since the year 2000 has been intramuscular fat (IMF). All birds (*n* = 520) were raised in three-story step cages (one bird/cage) under the same recommended environmental and nutritional conditions. Detailed information on animal management is described elsewhere (Liu et al., 2019; Liu et al., 2022).

The basal diet was formulated based on National Research Council (1994) requirements and the Feeding Standards of Chickens established by the Ministry of Agriculture, Beijing, China (2004). Venous blood was collected one day before slaughter into EDTA anticoagulation tubes and stored at −20°C for DNA extraction. In total, 520 chickens were slaughtered at the age of 98 days. After slaughtering, breast muscles were collected in liquid nitrogen and stored at −80°C until needed.

### Determination and analysis of aldehyde content

We previously generated VOC data from 519 breast muscles in JXY female chickens from a recent study using a gas chromatography‒mass spectrometry (GC-MS) method (Jin et al., 2021). In samples, 513 individuals had assay batch records. The relative aldehyde content data in 513 JXY chickens were adjusted using the GLM function in R software to correct for fixed effects that affect the phenotype and perform a significance test. The number of individuals with different traits used for GWAS was selected using the mean ± 2 standard deviations (Campbell and Robinson, 1993; Exilus et al., 2021; Grammatico-Guillon et al., 2021) from the value of their phenotypes: 467 (total aldehydes), 473 (hexanal), 475 (heptanal), 483 (benzaldehyde), 497 ((E,E)-2,4-nonadienal), 497 (octanal), 493 ((E)-2-decenal), 477 (nonanal), 498 (decanal), and 489 (octadecanal).

### DNA extraction and whole genome resequencing

Detailed information on genomic DNA extraction and sequencing was provided elsewhere (Liu et al., 2019; Liu et al., 2022). A DNA library of each sample was constructed, and at least 10 G of original sequencing data were obtained for each sample (raw data). Filtered pure reads were compared with the *Gallus gallus* (chicken) reference genome (GRCg6a: GCA_000002315.5) using the MEM mode of BWA software (Li and Durbin, 2009) (version 0.7.12) (http://bio-bwa.sourceforge.net). Then, Picard-tools (version 1.119) (https://broadinstitute.github.io/picard/) and SAMtools (Li et al., 2009) (version 1.9) (http://samtools.sourceforge.net/) were used to generate sorted BAM files. The Genome Analysis Toolkit (Mckenna et al., 2010) (GATK, version 4.0.2.1) was used to perform variant calling and variants for filtering. Details of the hard filtering standards for these populations have been described in previous studies (Liu et al., 2020).

### Genome-wide association studies

Clean DNA sequencing data were deposited in the Genome Sequence Archive (Wang et al., 2017) in the BIG Data Center (https://bigd.big.ac.cn/gsa/) (Members, 2019) under the accession numbers CRA002643 and CRA002650 (Liu et al., 2020; Tan et al., 2021). Data are publicly accessed at http://bigd.big.ac.cn/gsa. After quality control (miss 0.1, maf 0.05), 8,777,521 SNPs (510 birds) and 496,736 INDELs (516 birds) across 33 autosomes and sex chromosomes (Z and W) were retained. The number of individual phenotypes is shown in the “Determination and Analysis of Aldehyde Content” module. We initially performed univariate GWAS by applying a linear mixed model to account for associations between the relative content of aldehydes and effective SNPs, using GEMMA (Zhou and Stephens, 2012). The statistical model applied is:

y=Wα+xβ+u+ε

where y = the phenotypic values of n individuals, W = a covariance matrix used to control population structure, α = a vector of corresponding effects that comprises the intercept, x = marker genotypes, *β* = the effects of corresponding markers, u = a vector of random effects, and ε = a vector of residual errors. The top 1 PCA values (eigenvectors) were set as fixed effects in the mixed model. The *p*-value was corrected with a strict Bonferroni adjustment based on linkage disequilibrium (LD) pruning (Johnson et al., 2010). The sum of the independent LD blocks plus singleton markers was used to define the number of independent statistical comparisons (Bai et al., 2018; Wang et al., 2020). Finally, 361,337 independent SNPs and 26,421 independent INDELs were used to determine the *p*-value thresholds, including 5% genome-wide significance (SNPs: 1.38e-07, 0.05/361,337; INDELs: 1.89e-06, 0.05/26,421) and suggestive associations (SNPs: 2.77e-06, 1/361,337; INDELs: 3.78e-05, 1/26,421). GWAS Manhattan and QQ plots were produced using the CMplot and qqman packages in R v4.1.0. We performed a series of LD analyses to characterize causative SNPs and INDELs within strong LD regions by applying the solid spine algorithm in Haploview software version 4.2 (Barrett et al., 2005).

### Pathway analysis using gene ontology (GO) and kyoto encyclopedia of genes and genomes (KEGG)

Annotated genes nearest to or harboring significant SNPs and INDELs within a 100 kb range were identified as candidate genes where significant loci were located. Candidate genes in specific genomic regions were annotated using Variant Effect Predictor based on the GRCg6a (GCA_000002315.5) assembly supported by Ensembl (http://useast.ensembl.org/Gallus_gallus/Info/Index). The signaling pathway enrichment of candidate genes from significant variants was performed using “Gene-list Enrichment” in Kobas 3.0 (http://kobas.cbi.pku.edu.cn/) (Bu et al., 2021). Sankey diagrams were generated using the OmicShare tools (https://www.omicshare.com/tools).

### RNA extraction and sequencing

Total RNA was extracted from breast muscle tissue samples using TRIzol reagent (Invitrogen, Carlsbad, CA, United States). Detailed information on RNA extraction and sequencing was described elsewhere (Liu et al., 2019; Liu et al., 2022). Sequencing reads were mapped to GRCg6a (GCA_000002315.5) using the HISAT2 program (Sirén et al., 2014). To quantify the expression of each transcript, alignment results were analyzed by Cufflinks (v2.0.2) software (Ghosh and Chan, 2016). The FPKM of each gene was calculated based on the length of the gene and read count mapped to this gene. The FPKM data from 56 chicken breast muscles were used to perform the correlation with candidate gene expression and aldehyde content. The 56 birds with FPKM data, belonging to the 520 birds for genotyping, were collected from previous studies (Liu et al., 2019; Liu et al., 2021; Liu et al., 2022). The gene expression datasets, CRA004228, CRA001908, and CRA004003 were deposited in the BIG Data Center.

### Analysis of fatty acid data

The hydrolyzed fatty acid composition of breast muscle was determined by gas chromatography (GC) using a method from previous research (Liu et al., 2022). Based on chromatographic peak maps, fatty acid components in samples were determined by comparing peak times of standard products, and the percentage of each fatty acid was calculated using the peak area normalization method. C18:2n6c linoleic acid and C18:3n3 linolenic acid data were acquired from published data (Cui et al., 2022; Liu et al., 2022).

### Statistical analysis

VOCs were analyzed in Microsoft Excel 2016 (Microsoft Corp., Redmond, WA, United States). Data were expressed as the mean ± standard deviation (n = 513). Correlation analyses of VOCs with fatty acids and candidate gene expression were conducted in R-software (Version 4.1.0) with the “psych” by Spearman correlation methods. We extracted trait-related variants and explored differences in relative aldehyde content in meat samples with different genotypes using the Wilcoxon rank-sum test in R. Differences were considered significant at adjusted *p*-values < 0.05.

## Results

### Genome-wide association study for relative main aldehyde volatile components

**FIGURE 1:**
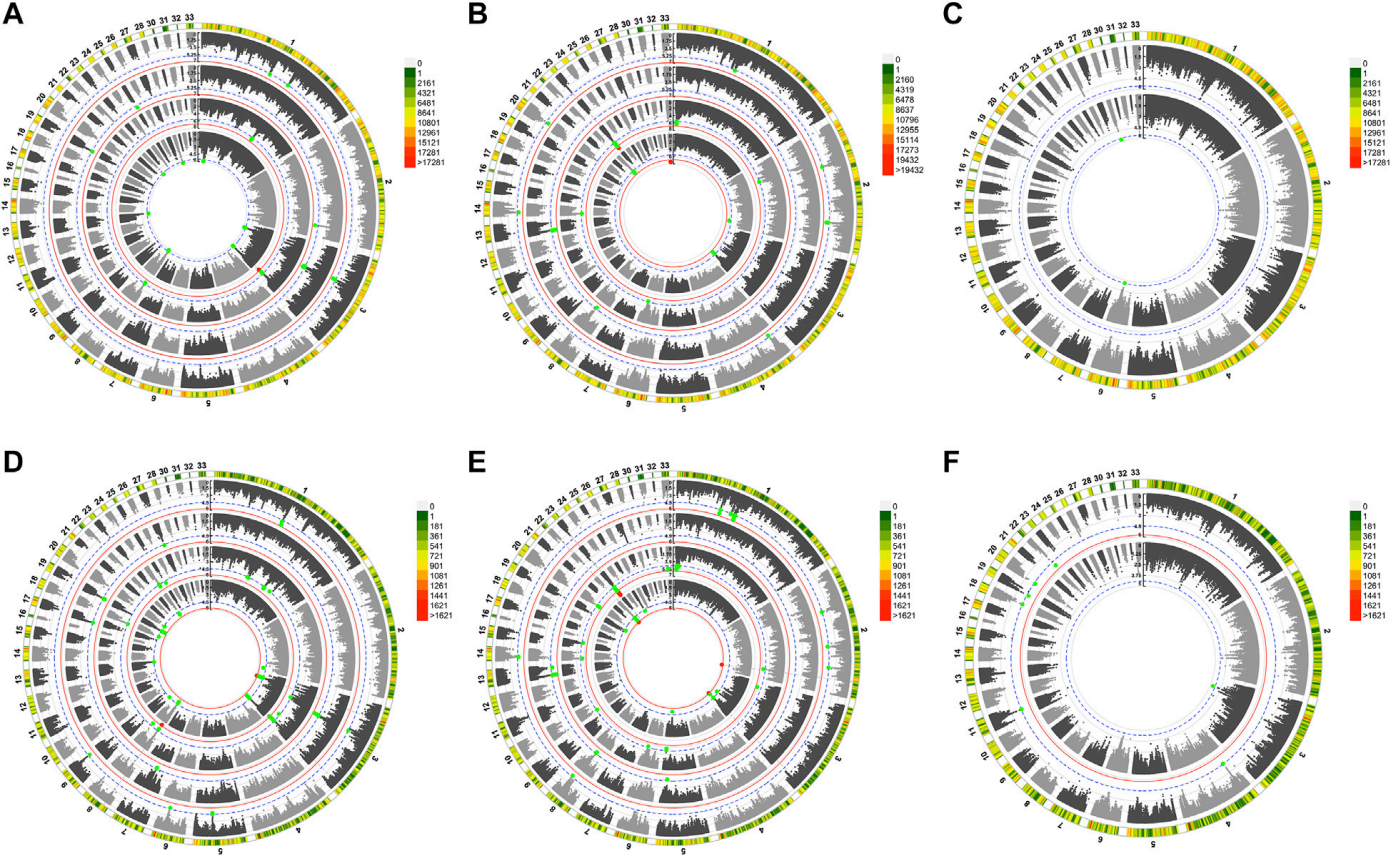
Manhattan plot landscapes of several aldehydes. The global view of Manhattan plot of GWAS on SNPs **(A,B,C)** INDELs **(D,E,F)** variants. **(A,D)** displayed from the inner to outer circle are the Manhattan plots of total aldehydes, hexanal, benzaldehyde, (E,E)-2,4-nonadienal, and Chromosome ideograms for *Gallus gallus*, respectively. **(B,E)** displayed from the inner to outer circle are the Manhattan plots of (E)-2-decenal, nonanal, decanal, octadecanal, and Chromosome ideograms for *Gallus gallus*, respectively. **(C,F)** displayed from the inner to outer circle are the Manhattan plots of heptanal, octanal, and Chromosome ideograms for *Gallus gallus*, respectively.

In total, nine aldehydes were detected in JXY chickens, including hexanal (C_6_H_12_O), heptanal (C_7_H_14_O), benzaldehyde (C_6_H_5_CHO), (E,E)-2,4-nonadienal (C_9_H_14_O), octanal (C_8_H_16_O), (E)-2-decenal (C_10_H_18_O), nonanal (C_9_H_18_O), decanal (C_10_H_20_O), and octadecanal (C_18_H_36_O), with hexanal being the most abundant (Table 1). A histogram of aldehyde content shows the aldehyde content distribution after eliminating extremes (Supplementary Figure S1). To study the genetic basis of aldehydes in chicken meat, we performed GWAS on the relative content of total aldehydes and several aldehydes with the data of SNP and INDEL, and identified signals associated with different aldehydes. The Manhattan plots (Figure 1) and Q-Q plots (Supplementary Figures S2–S3) of different aldehydes were shown.

**TABLE 1:** Mean relative aldehyde VOC content.

Traits	The relative content (Mean ± SD)
Total aldehydes	70.54 ± 20.39
Hexanal	43.87 ± 14.73
Heptanal	1.62 ± 0.59
Benzaldehyde	10.93 ± 4.52
(E,E)-2,4-Nonadienal	2.23 ± 1.04
Octanal	2.54 ± 1.13
(E)-2-Decenal	0.21 ± 0.13
Nonanal	7.82 ± 2.76
Decanal	0.09 ± 0.12
Octadecanal	1.22 ± 1.13

GWAS revealed 501 total variants (253 SNPs and 248 INDELs) for nine aldehyde VOCs, and total aldehydes related to chicken meat (Supplementary Table S1). Both analyses of two variant types co-localized to regions of the same chromosome, including chromosomes 3, 8 and 13 of total aldehydes; chromosomes 1, 3 and 8 of hexanal; chromosomes 3 and 19 of benzaldehyde; chromosomes 1 and 3 of (E,E)-2,4-nonadienal; chromosomes 2, 3 and 22 of (E)-2-decenal; chromosomes 1, 2, 6 and 23 of nonanal; chromosomes 8 and 13 of decanal; and chromosomes 1, 2 and 14 of octadecanal. Thus, multiple associated regions were identified for several aldehydes on chromosomes 1, 3 and 8. However, only a few potential variants were found for heptanal and octanal, with no potential SNP signal for octanal. Additionally, no overlapping regions were identified between SNP and INDEL variants for these two traits. Most significant variants were detected in noncoding regions (Supplementary Table S1).

### Kyoto encyclopedia of genes and genomes pathway analysis of candidate genes associated with different aldehydes

**FIGURE 2:**
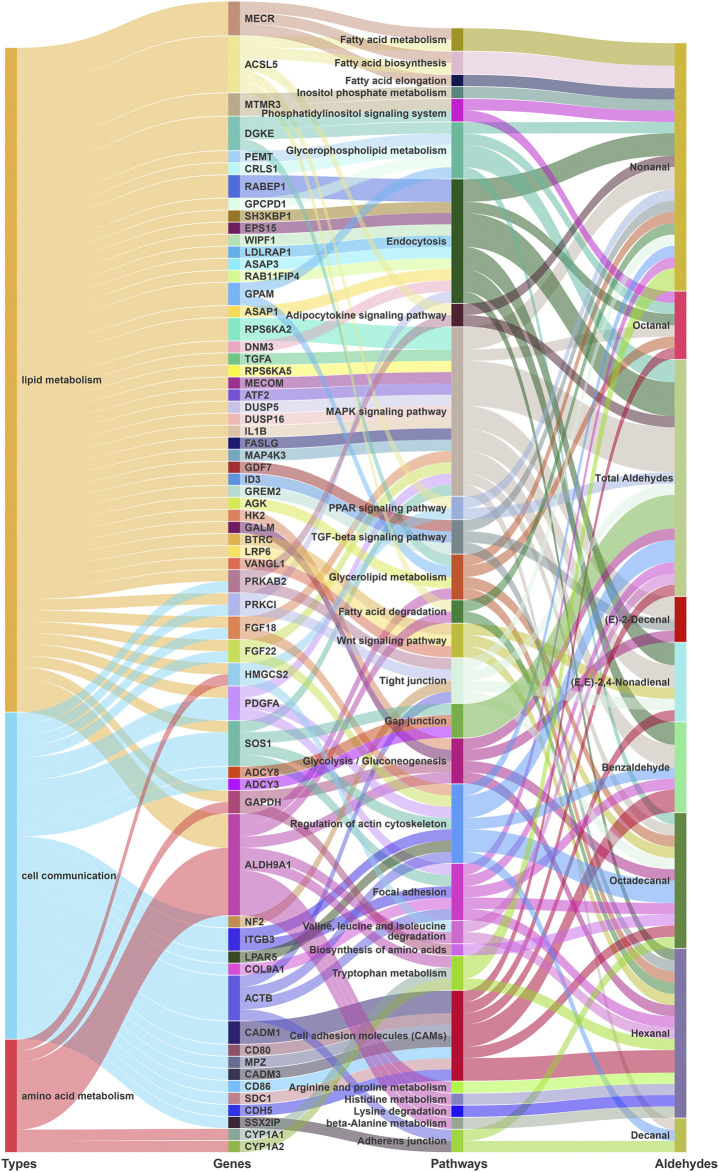
KEGG (kyoto encyclopedia of genes and genomes) pathway enrichment analyses for GWAS annotation. Sankey diagrams showing that potential genes from GWAS jointly participate in lipid metabolism, cell communication, and amino acid metabolism pathways.

We investigated 701 protein-coding genes in 100 kb regions flanking suggestive SNPs and INDELs for 10 traits, of which 511 known protein-coding genes were detected. Gene function annotations revealed that these candidate genes were mostly involved in biosynthetic and metabolic pathways of aldehyde precursors. For different fatty acid-derived aldehyde VOCs, several potential genes involved in lipid biosynthesis and degradation related pathways were detected (Figure 2; Supplementary Table S2). The candidate genes for more than four aldehydes were involved in the MAPK signaling pathway (8 kinds), endocytosis (6 kinds), glycerophospholipid metabolism (4 kinds), glycolysis/gluconeogenesis (4 kinds), and glycerolipid metabolism (4 kinds). The candidate genes of 2–3 aldehydes were involved in the TGF-β signaling pathway (3 kinds), PPAR signaling pathway (2 kinds), adipocytokine signaling pathway (2 kinds), fatty acid degradation (2 kinds), and Wnt signaling pathway (3 kinds) (Figure 2). For nonanal, myotubularin related protein 3 (*MTMR3*) was involved in inositol phosphate metabolism and phosphatidylinositol signaling. Also, mitochondrial trans-2-enoyl-CoA reductase (*MECR*) and acyl-CoA synthetase long chain family member 5 (*ACSL5*) were involved in fatty acid biosynthesis and fatty acid metabolism. Some genes were associated with different phenotypes. The ribosomal protein S6 kinase A2 (*RPS6KA2)* which was involved in the MAPK signaling pathway, was associated with both benzaldehyde and (E,E)-2,4-nonadienal. Rab GTPase-binding effector protein 1 (*RABEP1*), which was involved in endocytosis, was associated with both benzaldehyde and total aldehydes.

In addition, candidate genes were involved in pathways related to cell communications, including focal adhesion, cell adhesion molecules, tight junction, and regulation of actin cytoskeleton. Moreover, potential candidate genes were also involved in amino acid related pathways. Particularly for hexanal, aldehyde dehydrogenase 9 family member A1 (*ALDH9A1*) on chromosome 8 was enriched in multiple amino acid and lipid related metabolism and degradation processes. These genes involved in the aforementioned pathways may play important roles in aldehyde formation by regulating different pathways.

### Gene ontology analysis of candidate genes associated with different aldehydes

**FIGURE 3:**
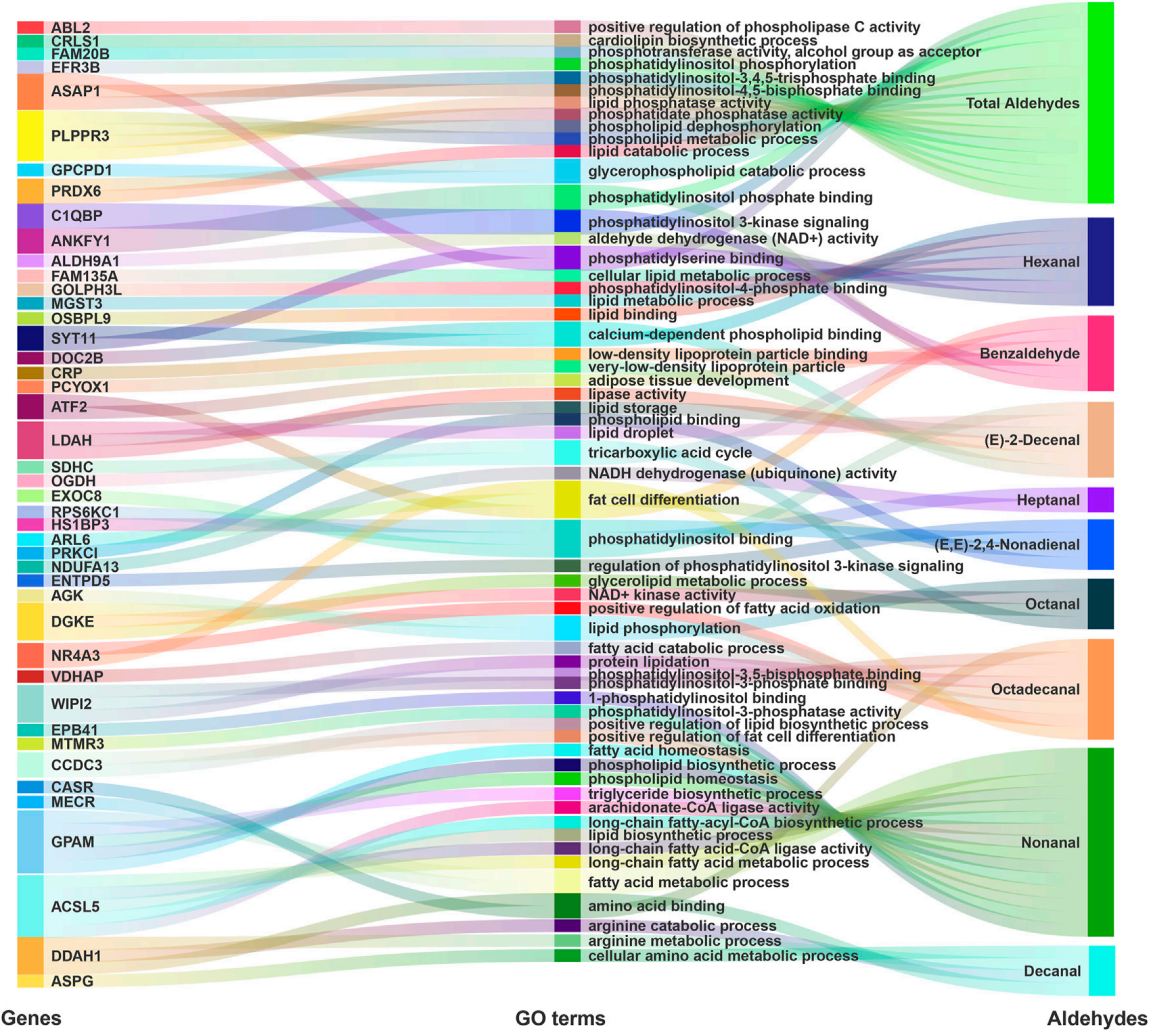
GO term enrichment analyses for GWAS annotation. Sankey diagrams showing that potential genes from GWAS participate in phospholipid and lipid related GO terms.

GO enrichment analyses indicated candidate genes related to lipid metabolism (Figure 3; Supplementary Table S3). Genes related to total aldehydes were involved in the glycerophospholipid catabolic process [peroxiredoxin 6 (*PRDX6*) and glycerophosphocholine phosphodiesterase 1 (*GPCPD1*)] and cardiolipin biosynthetic processes [cardiolipin synthase 1 (*CRLS1*)]. Also, phospholipid phosphatase related 3 (*PLPPR3*) was involved in multiple GO terms, including lipid phosphatase activity, phosphatidate phosphatase activity, phospholipid dephosphorylation, and phospholipid metabolic process. Complement C1q binding protein (*C1QBP*) was involved in phosphatidylinositol 3-kinase signaling. Moreover, ARF GTPase-activating protein 1 (*ASAP1*) was involved in phosphatidylinositol-3,4,5-trisphosphate binding, phosphatidylserine binding and phosphatidylinositol-4,5-bisphosphate binding.

Hexanal candidate genes were involved in phosphatidylinositol phosphate binding (synaptotagmin 11 (*SYT11*)), phosphatidylinositol-4-phosphate binding [Golgi phosphoprotein 3 like (*GOLPH3L*)], cellular lipid metabolic processes [family with sequence similarity 135 member A (*FAM135A*)], lipid metabolic processes [microsomal glutathione S-transferase 3 (*MGST3*)], and lipid binding [oxysterol binding protein like 9 (*OSBPL9*)]. For 2,4-nonadienal, candidate genes were involved in phospholipid binding [protein kinase C iota (*PRKCI*)], phosphatidylinositol binding [exocyst complex component 8 (*EXOC8*)], regulation of phosphatidylinositol 3-kinase signaling [ectonucleoside triphosphate diphosphohydrolase 5 (*ENTPD5*)], and fat cell differentiation [ADP ribosylation factor like GTPase 6 (*ARL6*)]. Moreover, for the aromatic aldehyde benzaldehyde, candidate genes were involved in phosphatidylinositol phosphate binding [ankyrin repeat and FYVE domain containing 1 (*ANKFY1*)] and phosphatidylinositol 3-kinase signaling (*C1QBP*). Notably, *ANKFY1* and *C1QBP* genes were associated with total aldehydes and benzaldehyde.

For Nonanal, candidate genes were involved in 1-phosphatidylinositol binding [erythrocyte membrane protein band 4.1 (*EPB41*)] and phosphatidylinositol-3-phosphatase activity [myotubularin related protein 3 (*MTMR3*)]. Furthermore, glycerol-3-phosphate acyltransferase, mitochondrial (*GPAM*) was involved in several GO terms, including fatty acid homeostasis, phospholipid biosynthetic process, phospholipid homeostasis, fatty acid metabolic process, and triglyceride biosynthetic process. *ACSL5* was also involved in arachidonate-CoA ligase activity, long-chain fatty-acyl-CoA biosynthetic process, long-chain fatty acid-CoA ligase activity, lipid biosynthetic processes, and long-chain fatty acid metabolic processes. For (E)-2-decenal, HCLS1 binding protein 3 (*HS1BP3*) was involved in phosphatidylinositol binding, and lipid droplet associated hydrolase (*LDAH*) was involved in lipase activity, lipid storage, and lipid droplets.

### Candidate loci and genes for total aldehydes

**FIGURE 4:**
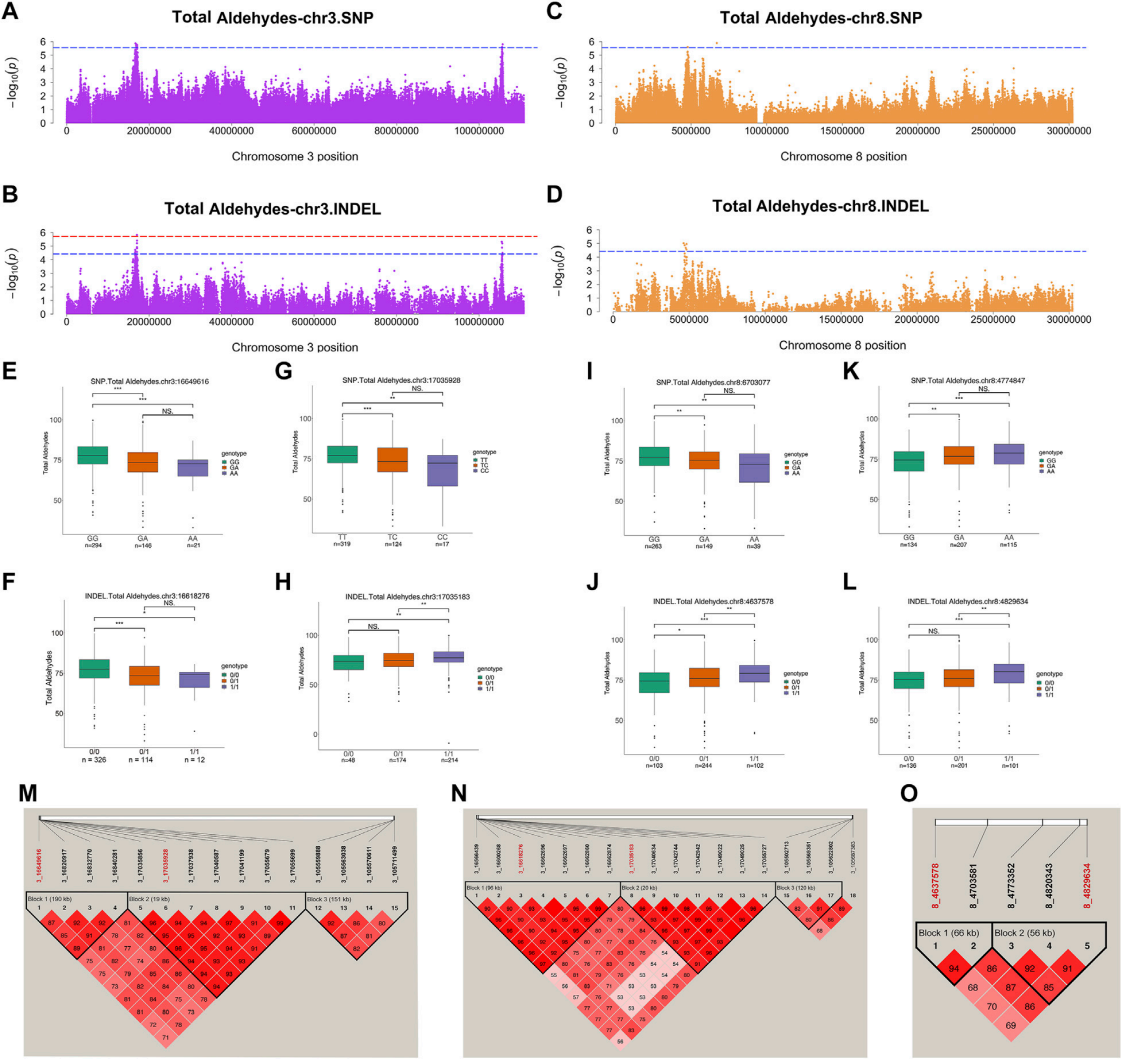
Genome-wide association studies for total aldehydes. **(A,B)** Manhattan plots for GWAS on SNPs **(A)** and INDELs **(B)** on chromosome 3 in total aldehydes. **(C,D)** Manhattan plots for GWAS on SNPs **(C)** and INDELs **(D)** on chromosome 8 in total aldehydes. The red, dashed line indicates the genome-wide significant threshold. The blue, dashed line indicates suggestive threshold. **(E–L)** Comparison of total aldehydes contents among genotypes within the total aldehydes -related locus on chromosome 3 **(E–H)** and 8 **(I–L)**. SNP at chr3:16649616 **(E)** was the highest signal in suggestive associations at chromosome 3. INDEL at chr3:16618276 **(F)** was nearest the SNP at chr3:16649616. Lipid-related genes were located adjacent to significant INDEL at chr3:17035183 **(H)** and suggestive SNP at chr3:17035928 **(G)**. SNPs at chr8:4774847 **(K)** and 6703077 **(I)** were the only two suggestive SNPs on chromosome 8. INDELs at chr8:4637578 **(J)** and 4829634 **(L)** were the 2 highest signals on chromosome 8 in the INDEL GWAS results. ***, **, * and NS. represent adjusted *p*-values < 0.001, <0.01, <0.05, and >0.05, respectively. **(M–O)** Haploview plot of linkage disequilibrium (LD) analysis of suggestive SNPs **(M)** and INDELs **(N)** located on chromosome 3. **(O)** Haploview plot of LD analysis of significant and suggestive INDELs located on chromosome 8. (LD values: D′). Red words represent locus annotated to lipid-related genes.

Among the aforementioned aldehydes, total aldehydes, hexanal, and nonanal had relatively strong signals. In addition, we analyzed loci where each trait co-localized to a chromosomal region in both variants. Genes from these lipid-related pathways were annotated and analyzed. The sum of total 9 aldehydes could represent the aldehydes in JXY chickens VOCs. For total aldehydes, SNPs and INDELs co-localized on same chromosome, including chromosome 3 and 8. These suggestive associations were detected in SNPs distributed over the chr3:16649616–17055699, 105559888–105711499, and chr8:4774847–6703077 regions (Figures 4A,C). Moreover, suggestive associations were detected in INDELs distributed over chr3:16566439–17055727, chr3:105502713–105687383, and chr8:4637578–4829634 regions (Figures 4B,D). There were no significant SNPs in total aldehydes GWAS results, but the SNP at chr3:16649616 was the highest signal in suggestive associations at chromosome 3. The chr3:16649616–16840281 region was a haploid block, where galactose mutarotase (*GALM*), SOS Ras/Rac guanine nucleotide exchange factor 1 (*SOS1*), *GPCPD1* and MAPK/ERK kinase kinase kinase 3 (*MAP4K3*) were detected (Table 2 and Figure 4M). The total aldehyde content in individuals with a GG wild genotype in the SNP at chr3:16649616 was significantly higher than that in individuals with an AA mutant genotype (Figure 4E). *GALM* was involved in glycolysis/gluconeogenesis and *SOS1* was involved in different pathways, including gap junction, MAPK signaling pathway, regulation of actin cytoskeleton and focal adhesion. *GPCPD1* was involved in glycerophospholipid metabolism and glycerophospholipid catabolic process (Figures 2, 3). Moreover, only one significant INDEL (chr3:17035183) and 6 suggestive INDELs were identified at chr3:17040634-17055727 in one block (Figure 4N). Seven suggestive INDELs at chr3:16566439-16662874 were in another block, so we chose one INDEL at chr3:16618276, which was nearest the SNP at chr3:16649616, for analysis (Figure 4N). *GALM* and *SOS1* were jointly located within 100 kb of the SNP at chr3:16649616 and the INDEL at chr3:16618276 (Table 2). Meanwhile, the wild type content was significantly higher than the mutant types in INDELs at chr3:16618276 (Figure 4F). For the significant INDEL at chr3:17035183, the total aldehyde content of individuals with wild genotypes was significantly lower than those with mutant genotypes (Figure 4H). Comparatively, individuals with a TT wild genotype in SNP at chr3:17035928 were significantly higher than individuals with a CC mutant genotypes (Figure 4G). *CRLS1*, TRNA methyltransferase 6 (*TRMT6*), and minichromosome maintenance complex component 8 (*MCM8*) were located adjacent to significant INDEL at chr3:17035183 and SNP at chr3:17035928 (Table 2). *CRLS1* was involved in glycerophospholipid metabolism (Figure 2).

**TABLE 2:** SNPs and INDELs with suggestive significance for total aldehydes, hexanal, and nonanal content.

Types	Trait	Chromosome	Position (bp)	Alt/Ref	*p*-value	Candidate genes^a^
SNP	Total aldehydes	3	16649616	G/A	1.34E-06	*HNRNPLL*, *ARHGEF33*, *GEMIN6*, *GALM*, *SRSF7*, *ATL2*, SOS1, *MORN2*
INDEL	Total aldehydes	3	16618276	TCA/T	1.81E-05	*HNRNPLL*, *ARHGEF33*, *GEMIN6*, *GALM*, *EIF4A3*, ALT2, *SRSF7*, *SOS1*, *MORN2*
SNP	Total aldehydes	3	17035928	T/C	1.72E-06	*TRMT6*, *MCM8*, *CRLS1*, *C3H1orf95*, *PARP1*, *CHGB*, *SHLD1*
INDEL	Total aldehydes	3	17035183	AT/T	1.50E-06	
SNP	Total aldehydes	8	4774847	G/A	2.51E-06	*SUCO*, *PRKAB2*, *PLPP6*, *FAM78B*, *PRDX6*, *FASLG*, *TADA1*
SNP	Total aldehydes	8	6703077	G/A	1.26E-06	*TOR3A*, *FAM20B*, *RALGPS2*, *ANGPTL1*, *ABL2*
INDEL	Total aldehydes	8	4637578	T/TG	9.48E-06	*PRKAB2*, *REG4*, *HMGCS2*, *PLPP6*, *FAM78B*, *PRDX6*, *NOTCH2*, *TADA1*, *SEC22B*
INDEL	Total aldehydes	8	4829634	A/AT	1.10E-05	*SUCO*, *DNM3*, *FASLG*, *PIGC*
SNP	Hexanal	3	101376953	A/G	7.53E-08	*LAPTM4A*, *MATN3*, *WDR35*, *TTC32*
INDEL	Hexanal	3	101539230	TG/T	1.63E-06	*SDC1*, *PUM2*
INDEL	Hexanal	3	36279875	CT/C	3.25E-05	*RGS7*, *GREM2*
INDEL	Hexanal	3	82837851	A/ACTAT	1.96E-05	*FAM135A*, *COL9A1*
INDEL	Hexanal	8	5844087	C/CA	1.17E-06	*LRRC52*, *MGST3*, *ALDH9A1*, *UCK2*, *DARS2*, *NUF2*, *KLHL20*, *CENPL*, *TMCO1*
SNP	Hexanal	8	5847470	C/A	1.14E-06	
SNP	Nonanal	23	2466221	T/C	9.31E-08	*TAF12*, *MTFR1L*, *MAN1C1*, *LDLRAP1*, *TMEM57*, *SYF2*, *YTHDF2, RHCE*, *ID3*, *RSRP1*, *GMEB1*, *TMEM50A*
INDEL	Nonanal	23	2467306	G/GAC	1.14E-06	
SNP	Nonanal	6	27634814	T/C	2.20E-06	*GPAM*, *TECTB*, *ZDHHC6*, *ACSL5*, *VTI1A*
INDEL	Nonanal	23	2994896	C/CT	3.02E-05	*PTPRU*, *MECR*, *SRSF4*

a Genes located within 100 kb upstream or downstream of the suggestively associated SNPs and INDELs.

On chromosome 8, two suggestive SNPs at chr8:4774847 and 6703077 were detected, and five INDELS from 4637578 to 4829634 were located in two blocks (Figure 4O). Thus, representatives in different haploid blocks were used to analyze relationships between genotype and content. These results showed that, except for the SNP at chr8:6703077 (Figure 4I), the total aldehyde content of individuals with wild-type genotypes was significantly lower than in individuals with mutant genotypes in suggestive SNPs and INDELs on chromosome 8 (Figures 4J–L). *PRDX6*, protein kinase AMP-activated non-catalytic subunit beta 2 (*PRKAB2*), and Fas ligand (*FASLG*) were jointly located within 100 kb of suggestive SNP at chr8:4774847 and INDELs at chr8:4637578-4829634 (Table 2). These genes were involved in glycerophospholipid catabolic process, adipocytokine signaling pathway and MAPK signaling pathway, respectively (Figures 2, 3). To further explore the relationships between potential genes and aldehyde content, we performed a correlation analysis with gene expression and aldehyde content. *TRMT6* (r = 0.37, *p* value = 0.0055) and *MCM8* (r = 0.42, *p* value = 0.0013) expression was significantly positively correlated with the total aldehydes content. *ASAP1* was annotated on INDEL at chromosome 2, and its expression was significantly negatively correlated with total aldehyde content (r = −0.31, *p* value = 0.021) (Supplementary Table S4). Furthermore, total aldehydes were significantly positively correlated with C18:2n6c linoleic acid (Table 3).

**TABLE 3:** Correlation coefficients of total aldehydes, hexanal, and nonanal with C18:2n6c and C18:3n3, respectively.

Traits	The relative content (Mean ± SD)	Correlation coefficient of C18:2n6c	Correlation coefficient of C18:3n3
Total aldehydes	70.54 ± 20.39	0.10*	0.07
Hexanal	43.87 ± 14.73	0.15**	0.13**
Nonanal	7.82 ± 2.76	−0.03	−0.04

*, Correlation analysis significance *p*-value < 0.05; **, Correlation analysis significance *p*-value < 0.01.

### Candidate loci and genes for hexanal

**FIGURE 5:**
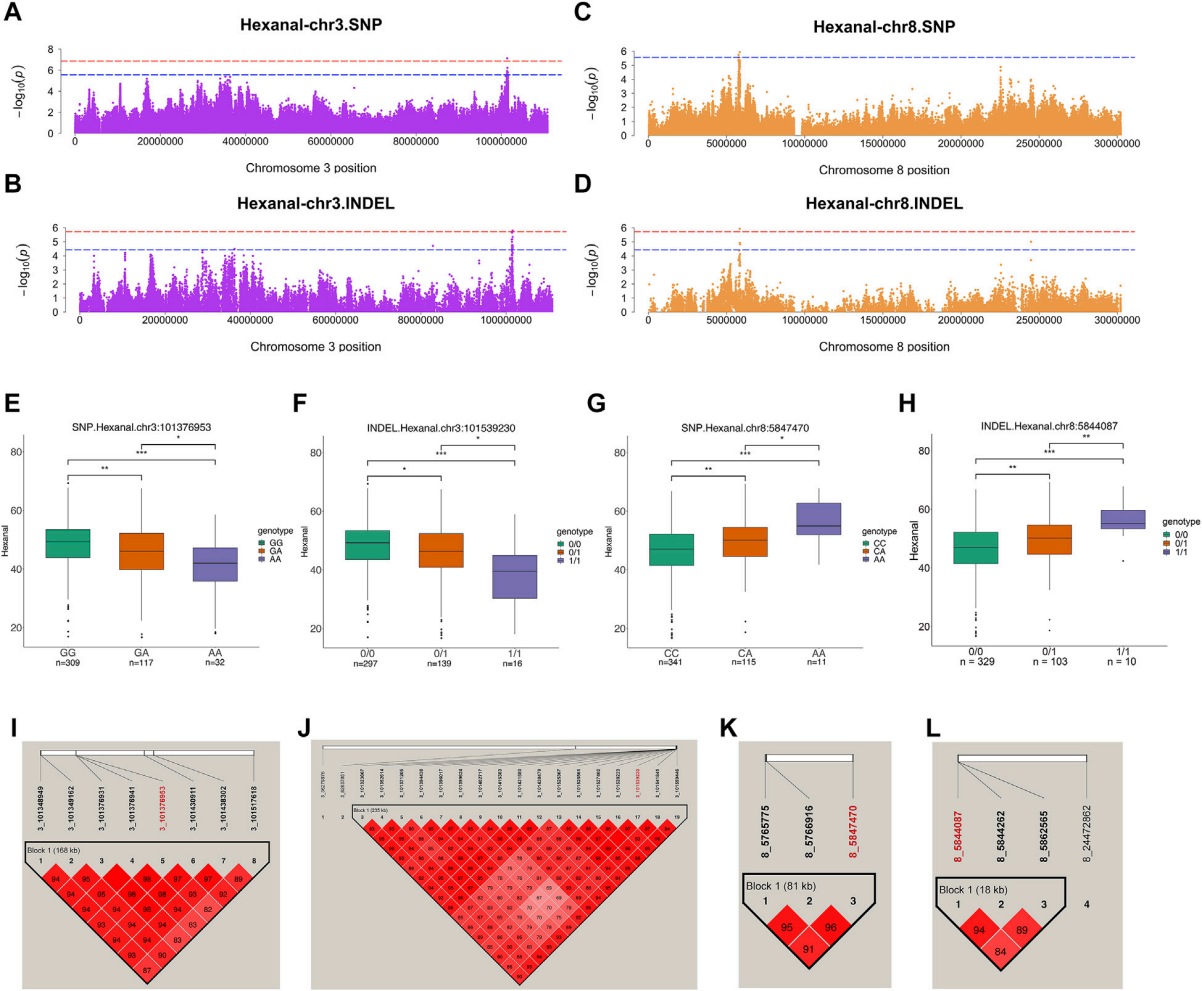
Genome-wide association studies for hexanal. **(A,B)** Manhattan plots for GWAS on SNPs **(A)** and INDELs **(B)** on chromosome 3 in hexanal. **(C,D)** Manhattan plots for GWAS on SNPs **(C)** and INDELs **(D)** on chromosome 3 in hexanal. The red, dashed line indicates the genome-wide significant threshold. The blue, dashed line indicates suggestive threshold. **(E–H)** Comparison of hexanal contents among genotypes within the hexanal-related locus on chromosome 3 **(E,F)** and 8 **(G,H)**. One significant SNP [chr3:101376953 **(E)**] and two INDELs chr3:101539230 **(F)** and chr8:5844087 **(H)** were identified. The SNP at the chr8:5847470 **(G)** was the highest signal in suggestive associations. ***, **, * and NS. represent adjusted *p*-values < 0.001, <0.01, <0.05, and >0.05, respectively. **(I,J)** Haploview plot of LD analysis of suggestive SNPs **(I)** and INDELs **(J)** located on chromosome 3. **(K,L)** Haploview plot of LD analysis of suggestive SNPs **(K)** and INDELs **(L)** located on chromosome 8. (LD values: D′). Red words represent locus annotated to lipid-related genes.

Hexanal was the most abundant aldehyde in chickens and was analyzed further. SNPs and INDELs for hexanal co-localized on the same chromosome, including chromosomes 1, 3, and 8. The suggestive associations were detected in SNPs distributed over the chr1:125687594–126303615, chr3:101348949–101517618, and chr8:5765775–5847470 regions (Figures 5A,C). Moreover, suggestive associations were detected in INDELs distributed over chr1:81289516–141532200, chr3:101323667–101559446, and chr8:5844087–5862565 regions (Figures 5B,D). One significant SNP (chr3:101376953) and two INDELs (chr3:101539230 and chr8:5844087) were identified from hexanal GWAS (Table 2; Supplementary Table S1A). One significant SNP at chr3:101376953 and 7 suggestive SNPs at chr3: 101348949-101517618 were located in one block (Figure 5I). Meanwhile, one significant INDEL at chr3:101539230 and 16 suggestive INDELs at chr3:101323667-101559446 were also located in one block (Figure 5J). For the significant SNP at chr3:101376953 and INDEL at chr3:101539230, the total aldehyde content of individuals with wild-type genotypes was significantly higher than that in individuals with mutant genotypes (Figures 5E,F). Syndecan 1 (*SDC1*) and pumilio2 (*PUM2*) were located within 100 kb of suggestive SNP (Supplementary Table S1E) and significant INDEL on chromosome 3 (Table 2). *SDC1* was involved in cell adhesion molecules. *PUM2* expression was significantly negatively correlated with hexanal content (r = −0.28, *p* value = 0.04) (Supplementary Table S4). Additionally, Gremlin-2 (*GREM2*), alpha-1(IX) collagen chain (*COL9A1*), and *FAM135A* were identified on chromosome 3 (INDELs: 36279875, 82837851), and were involved in the TGF-β signaling pathway, focal adhesion and cellular lipid metabolic process, respectively (Table 2; Figures 2, 3).

On chromosome 8, suggestive SNPs at chr8:5765775–5847470 were located in the one block (Figure 5K). One significant INDEL at chr8:5844087 and suggestive INDELs at chr8:5844262-5862565 were also in one block (Figure 5L). The SNP at chr8:5847470 was the highest signal in suggestive associations (Table 2; Supplementary Table S1A). Comparatively, for the SNP at chr8:5847470 and the INDEL at chr8:5844087, individuals with wild-type genotypes had a lower hexanal content than those with mutant genotypes (Figures 5G,H). *MGST3* and *ALDH9A1* were identified within 100 kb of SNPs at chr8:5847470 and INDELs at chr8:5844087 (Table 2). In addition, *MGST3* was involved in lipid metabolic process and *ALDH9A1* was involved in fatty acid degradation and several amino acid metabolism related pathways (Figure 2). Similar to total aldehydes, hexanal was positively associated with C18:2n6c linoleic acid and C18:3n3 linolenic acid (Table 3).

### Candidate loci and genes for nonanal

**FIGURE 6:**
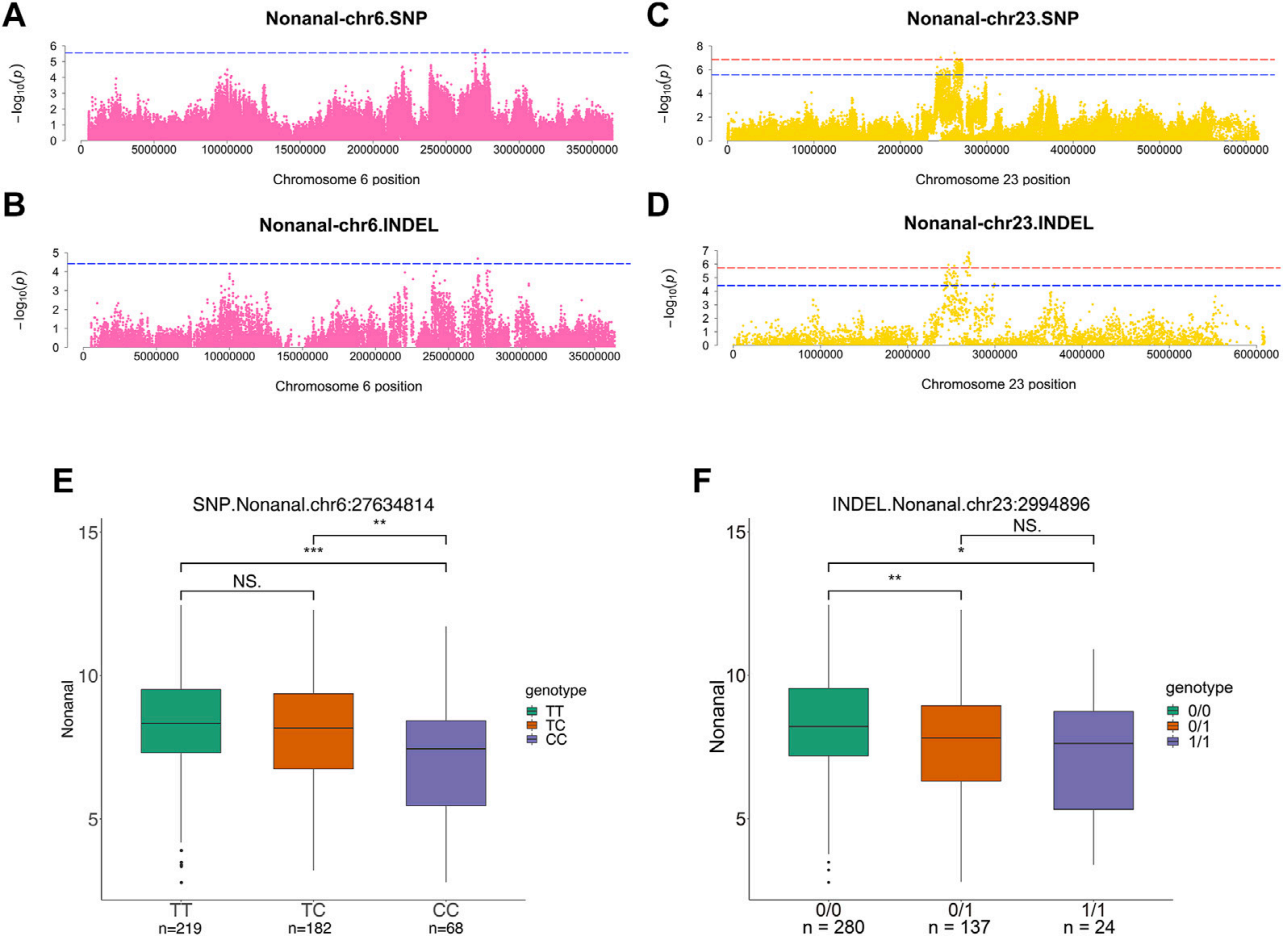
Genome-wide association studies for nonanal. **(A,B)** Manhattan plots for GWAS on SNPs **(A)** and INDELs **(B)** on chromosome 6 in nonanal. **(C,D)** Manhattan plots for GWAS on SNPs **(C)** and INDELs **(D)** on chromosome 23 in nonanal. The red, dashed line indicates the genome-wide significant threshold. The blue, dashed line indicates suggestive threshold. **(E,F)** Comparison of nonanal contents among genotypes within the nonanal-related locus. Lipid-related genes were located adjacent to suggestive SNPs at chr6:27634814 **(E)** and suggestive INDELs at chr23:2994896 **(F)**. ***, **, * and NS. represent adjusted *p*-values < 0.001, <0.01, <0.05, and >0.05, respectively.

For nonanal, the results showed that the largest number of significant variable loci. SNPs and INDELs co-localized on the same chromosomes, including chromosomes 1, 6, and 23. Suggestive associations were detected in SNPs distributed over the chr1:2346912–2840963, chr6:27634814–27658332, and chr23:2420497–2719201 regions (Figures 6A,C). Moreover, suggestive associations were detected in INDELs distributed over the chr1:2402493–2816746, chr6:27002268, and chr23:2422240–2994896 regions (Figures 6B,D). There were four significant SNPs and 16 INDELs at chromosome 23 from the nonanal GWAS (Supplementary Table S1A). Alpha1,2-mannosidase subtype IC (*MAN1C1*), low-density lipoprotein receptor adapter protein 1 (*LDLRAP1*), inhibitor of differentiation 3 (*ID3*), and glucocorticoid modulatory element-binding protein 1 (*GMEB1*) were identified within 100 kb of the significant SNP at chr23: 2466221 and INDEL at chr23:2467306 (Table 2). *ASAP3* was located within 100 kb of the suggestive SNPs at chr23:2420497-2436379 and suggestive INDELs at chr23:2422240-2450218. Furthermore, *MAN1C1* and *ID3* were involved in metabolic pathways and the TGF-beta signaling pathway, respectively. *LDLRAP1* and *ASAP3* were both involved in endocytosis. Thus, *GMEB1* expression was significantly positively correlated with nonanal content (r = 0.31, *p* value = 0.019) (Supplementary Table S4).

For suggestive SNPs at chr6:27634814 and suggestive INDELs at chr23:2994896, the wild-type content was higher than mutant genotypes (Figures 6E,F). *GPAM* and *ACSL5* were located within 100 kb of the SNP at chr6: 27634814. *MECR* was located within 100 kb of INDEL at chr23:2994896. Moreover, *GPAM* was involved in glycerophospholipid metabolism and glycerolipid metabolism. *MECR* and *ACSL5* were involved in fatty acid related pathways (Figure 2).

## Discussion

Understanding the genetic basis of aldehyde VOCs in chickens is essential for improving meat flavors. JXY chicken is an important resource in breeding quality chicken and is renowned for its excellent meat quality and delicious flavor, with its meat rich in major VOC components (Jin et al., 2021). A previous analysis indicated that the relative content of total aldehydes in JXY chickens was higher than that in Tiannong Partridge chickens and Wenchang chickens (Jin et al., 2021). For the JXY chicken, artificial selection for increased IMF content in pectoral muscles has been undertaken since 2000, and selection for breast muscle IMF could lead to desirable changes in meat quality (Zhao et al., 2007). We performed GWAS in JXY chickens to identify genetic markers and genes associated with 10 aldehyde traits to provide a scientific basis for improving flavor in high-quality chickens. In addition, providing a comprehensive analysis of multiple local chicken breeds for future studies will make our results more representative. Multiple lipid metabolism related genes were associated with different aldehydes using functional analyses, especially fatty acid and phospholipid related genes. Furthermore, loci in several important aldehydes (total aldehydes, hexanal, and nonanal) with strong associations were further analyzed. In particular, total aldehydes are the sum of aldehydes detected in JXY chickens, and related loci could be used as representative loci for aldehydes. We compared the relative aldehyde content among genotypes in key aldehyde VOCs related loci, and analyzed the mRNA expression of candidate genes associated with these aldehydes. We identified several potential sites and genes associated with chicken meat aldehyde VOC traits.

Although the volatile landscape in chickens has been characterized in several studies (Jayasena et al., 2013; Takakura et al., 2014; Fan et al., 2018; Feng et al., 2018; Jin et al., 2021), the genetic basis of VOC variants among genotypes remains unknown. Since VOCs in meat products are produced by several reactions via heat treatment, these substances are trace-like in terms of detection. This may explain why the signal strength of gene loci for trace content substances produced by several reactions was not very strong. We identified molecular markers for aldehydes using GWAS, and showed that a few genomic regions were involved in the variant of VOC content in chicken meat. GWAS was previously used to analyze VOC content in tomatoes (Tieman et al., 2017; Li et al., 2020), apples (Larsen et al., 2019), blueberries (Ferrão et al., 2020), and cacao beans (Colonges et al., 2022). For fatty acid derivative VOCs (aldehydes, alcohols, and methyl ketones) in fruit, several enzymes involved in lipid metabolism were identified. Fatty acid synthesis and degradation can have a major impact on downstream volatile synthesis, as indicated by tomato studies (Howe and Schilmiller, 2002; Garbowicz et al., 2018). We also identified genes involved in aldehyde lipid metabolism in chicken meat. Moreover, few studies have explored genomic variability with respect to the sensory qualities of meat products. A study indicated that some QTLs affecting several sensory (flavor and texture) of dry-cured hams from pigs were detected (Pena et al., 2013). Also, the several functional candidate genes involved in the biochemical processes that shaped flavor and textural attributes.

Similar to other meats, the flavor development in poultry meat is partly attributed to the lipid content (Jayasena et al., 2013). Several hundred volatile compounds are generated in cooked meat via lipid degradation, primarily via the oxidation of fatty acid components of lipids. Such compounds include, aliphatic hydrocarbons, aldehydes, alcohols, ketones, esters, carboxylic acids, and some aromatic hydrocarbons (Jayasena et al., 2013). Aldehydes are vital odor compounds in chicken meat and soup due to their lower odor thresholds and higher concentrations, which are mainly derived from fatty acid degradation and oxidation. Hexanal had the highest relative content in chicken meat and is well known in the food flavor industry for its grass odor/flavor (Jin et al., 2021). Also, (E,E)-2,4-nonadienal is a well-known volatile constituent in cooked chicken, with fatty and chicken notes, and is considered a key contributor to chicken aroma (Fan et al., 2018; Feng et al., 2018). Hexanal and (E,E)-2,4-nonadienal may be formed from the autoxidation of n-6 PUFAs such as linoleic acid and arachidonic acid (Fan et al., 2018; Feng et al., 2018). Octanal (citrus, fatty, and fruity) and nonanal (floral, green, and fatty) are generated from n-9 PUFA oxidation (Tanimoto et al., 2015; Qi et al., 2017). Moreover, benzaldehyde is the most commonly used aromatic aldehyde in the food industry, and has a cherry-like and bitter almond odor (Chen et al., 2020). In addition to lipid degradation and oxidation, the Maillard reaction also contributes to aldehyde production, e.g., nonanal and benzaldehyde are the two main aldehydes found in all Maillard reaction products (Wang et al., 2019). (E)-2-Decenal has a fatty, tallowy odor, and decanal has a green, fatty, citrus odor (Fan et al., 2018; Feng et al., 2018). In summary, the reaction of different kinds of fatty acid components of lipids is mainly responsible for the formation of different aldehydes in chicken meat. Saturated and unsaturated aldehydes play a vital role in all cooked meat aromas. However, the lower correlation between aldehydes and fatty acid content may be due to the fact that hydrolyzed fatty acids were collected in this study rather than free fatty acid hydrolysis, and implied that free fatty acids may contribute more to flavor. Additionally, our previous study indicated that several suggestive SNPs associated with PUFAs were identified by GWAS, including 24 SNPs located on GGA1 and GGA2 for linoleic acid, and three SNPs located on GGA3 (chr3:88650400, 88721989, and 88744336) for linolenic acid (Liu et al., 2022). Although loci on chromosome 3 were associated with linolenic acid, they were not in the same region compared to the aldehyde GWAS results. This might be due to the fact that flavor volatile compounds are generated through heat-induced complex reactions of various flavor precursors such as fatty acids and amino acids, making the results different from those of fatty acid phenotypes.

The lipid degradation of fatty acids contributes to the largest number of odorants in chicken broth and meat, and includes aliphatic aldehydes. In our study, we identified several candidate genes involved in fatty acid degradation, metabolism and biosynthesis, and phospholipid related pathways and GO terms, which was consistent with the principle of the production of aldehyde volatiles from meat, as fatty acids are one of the most critical flavor precursors for meat. For example, *MECR*, as a potential gene for nonanal, was involved in fatty acid biosynthesis, fatty acid metabolism and fatty acid elongation. *MECR* was shown to improve the transcriptional activation of PPARs to regulate feeding and physical activity (Kim et al., 2014; Cheng et al., 2020). Also, *ACSL5,* associated with nonanal, is an isozyme that converts free long-chain fatty acids into fatty acyl-CoA esters, and has key roles in lipid biosynthesis and fatty acid degradation. The *ACSL5* SNP was associated with rapid weight loss in obese females (Teng et al., 2009). Previous studies of the liver indicated that *ACSL5* over-expression promoted DAG and TG synthesis from fatty acids (Parkes et al., 2006; Bu and Mashek, 2010; Yan et al., 2015). However, *ACSL5* may function differently depending on the tissue, and higher *ACSL5* mRNA levels reflected lower total cholesterol and triglyceride levels in skeletal muscle (Adamo et al., 2007; Bu and Mashek, 2010; Zhang et al., 2018). In the present study, *ACSL5* expression was not significantly correlated with nonanal content in breast muscle.

The relative content of hexanal was the highest of the aldehyde VOC class among the JXY, Tiannong Partridge, and Wenchang chickens (Jin et al., 2021). Our previous study in Tiannong Partridge chickens showed that the differentially expressed genes between the high and low hexanal content groups were significantly enriched in the PPAR signaling pathway, glycerophospholipid metabolism, MAPK signaling pathway, fatty acid biosynthesis, fatty acid degradation, etc. (Jin et al., 2022). In this study, hexanal candidate genes were also involved in multiple fatty acid and amino acid related pathways, and lipid related GO terms. We observed that *GALM* and *ALDH9A1* were associated with total aldehydes and hexanal, respectively. They were both involved in metabolic pathways and glycolysis/gluconeogenesis. A previous study reported lower *GALM* expression related to glycometabolism and higher *ALDH9A1* expression related to lipolysis in Yunling cattle, with a higher proportion of saturated fatty acids, PUFAs, and short-chain fatty acids (Zhang et al., 2018). Additionally, this study showed that *GALM* was associated with meat quality traits in beef cattle. A previous study reported that hexanal levels predicted the development of oxidation flavors in different types of uncured delicatessen meat products (Bak and Richards, 2021). In our study, *ALDH9A1* was one of the candidate genes significantly associated with hexanal, and was involved in several amino acid and fatty acid related pathways. Previous reports also showed that *ALDH9A1* was involved in cell functions such as aldehyde detoxification and fatty acid metabolism (Zhang et al., 2014). Additionally, aldehyde dehydrogenases oxidized aldehydes to corresponding carboxylic acids using either NAD or NADP as coenzymes (Muzio et al., 2012), therefore we speculate that *ALDH9A1* may participate in the formation and degradation of hexanal. PUFAs are oxidized and decomposed to yield volatile compounds (Fan et al., 2018). We observed that candidate genes were dominated by lipid biosynthesis and degradation related pathways, including *ACSL5*, *MECR*, *ALDH9A1*, etc.

Lean meat contains intramuscular triglycerides and structural phospholipids. Previous studies indicated that phospholipids have more important roles in the formation of aroma volatiles during meat cooking than triglycerides, probably due to a much higher proportion of unsaturated fatty acids in phospholipids (Mottram, 1998; Jayasena et al., 2013). Additionally, the glycerophospholipid metabolism pathway was associated with chicken meat flavor (Zhang et al., 2022). In our study, several candidate genes were involved in phospholipid related pathways, consistent with previous reports. For example, *CRLS1* and *GPCPD1* were involved in glycerophospholipid metabolism and were associated with total aldehyde content. *CRLS1* silencing also reduced cardiolipin levels (Maekawa et al., 2019). *GPCPD1* appeared to catalyze sn-glycero-3-phosphocholine to sn-glycerol 3-phosphate, which are two metabolomic biomarkers involved in the discrimination of meat quality characteristics in chicken breeds (Zhang et al., 2022). *GPAM* was identified as a potential gene associated with nonanal. The *GPAM* could promote triglyceride synthesis in the fat metabolism pathway (Li et al., 2019). Different *GPAM* genotypes were significantly associated with the fatty acid composition of IMF in bovines, and its polymorphic loci were associated with higher milk fat percentages in dairy cattle (Yu et al., 2017; Yu et al., 2021). Furthermore, *ASAP1* was associated with total aldehydes and was involved in phosphatidylinositol-3,4,5-trisphosphate, phosphatidylserine, and phosphatidylinositol-4,5-bisphosphate binding. A previous study indicated that *ASAP1* was located on bovine chromosome 14, where QTLs for fat thickness, yield grade, and marbling were reported (Casas et al., 2003), and a novel association was also identified between an *ASAP1* SNP and shear force measured at 24 h post mortem in Nelore cattle (Tizioto et al., 2012). Thus, *ASAP1* may be involved in the regulation of membrane trafficking and cytoskeleton remodeling, or it may have roles in tissue differentiation related to adipocytes. We found that *ASAP1* was negatively correlated with total aldehydes content. These genes may help regulate fatty acid deposition in phospholipids to influence flavor content.

In our study, genes in other lipid-related and cell communication pathways were identified. Candidate genes were also involved in the endocytosis of different aldehydes. Endocytosis is essential for material transport, especially for large molecules or those that cannot easily penetrate the cell plasma membrane (Doherty and McMahon, 2009). A previous report showed that fatty acids were transported into cells via CD36-mediated endocytosis (Hao et al., 2020). *ASAP1* and *LDLRAP1* was also involved in endocytosis. A study showed that *LDLRAP1* played a central role in adipocyte biology and participated in adipose metabolism and regulation of insulin sensitivity and adipose metabolic processes (Leigh et al., 2022). Additionally, most aldehydes were associated with several MAPK pathway genes, except hexanal. It was previously reported that MAPK and TGF-β signaling pathways interacted with the PPAR pathway to regulate lipid metabolism during lipogenesis processes in chickens (Cui et al., 2012; Ma et al., 2021). Also, other studies reported the indirect effects of lipids on MAPK signaling via endocytosis regulation (Anderson, 2006). We observed that potential genes involved in MAPK and endocytosis were associated with different aldehydes. *RPS6KA2* was involved in MAPK signaling and associated with benzaldehyde and (E,E)-2,4-nonadienal, while *MAP4K3* was associated with total aldehydes. A previous study reported that *MAP4K3* mutant flies had low lipid reserves (Bryk et al., 2010). *ID3* was associated with nonanal and involved in TGF-β signaling pathways. *SDC1* was involved in cell adhesion molecules (CAMs) and was associated with hexanal. A study in mice suggested that Sdc1 was required to maintain intradermal fat and confirmed that the protein was expressed and induced during adipocyte differentiation (Kasza et al., 2014). In addition, some candidate genes have been reported to be related to lipids, and their expression levels in the present study were significantly correlated with flavor content. *TRMT6* was one of the significantly associated candidates for total aldehyde content and its expression was positively correlated with the content. The study showed that the m1A methyltransferase complex (TRMT6 and TRMT61A) could increase PPARδ translation, which in turn triggers cholesterol synthesis to activate Hedgehog signaling in human hepatocellular carcinoma cell lines (Wang et al., 2021). Meanwhile, PPARδ stimulates lipid and glucose utilization by increasing mitochondrial function and fatty acid desaturation pathways (Montaigne et al., 2021). This gene might affect lipid and glucose utilization and thus flavor substance content by regulating the expression of PPARδ. *PUM2* was one of the significantly associated candidates for hexanal content and its expression was negatively correlated with the content. A previous study showed that depletion of PUM2 blocks MSC adipogenesis and enhances osteogenesis (Lee et al., 2020). *GMEB1* was associated with nonanal content and its expression was positively correlated with the content. *GMEB1* is a transcription factor that is essential for parvovirus DNA replication and modulates the transactivation of the glucocorticoid receptor (Kotsaris et al., 2020). *GMEB1* has been shown to have a significant association with Child Obesity trait (Song et al., 2022). We speculate that these genes regulate fatty acids, which are aldehyde precursors. However, due to the complexity of volatile substances produced by heating and other methods, correlations between gene expression and aldehyde content may be weak. Although potentially viable candidate genes were identified, we did not detect causal polymorphisms in genes, therefore, functional validation studies are required to confirm our hypotheses.

## Conclusion

In this study, GWAS analyses of SNPs and INDELs associated with flavor-presenting aldehydes were conducted, thus revealing potential loci and candidate genes associated with total aldehydes, hexanal, heptanal, benzaldehyde, (E,E)-2,4-nonadienal, octanal, (E)-2-decenal, nonanal, decanal, and octadecanal in Chinese local chicken meat. The candidate genes involved in lipid metabolism were identified, particularly fatty acid-related pathways, and those pathways associated with aldehyde VOCs in chicken meat; these genes included *GALM*, *MAP4K3*, *GPCPD1*, *RPS6KA2, CRLS1*, *ASAP1*, *TRMT6*, *SDC1*, *PUM2*, *ALDH9A1*, *MGST3*, *GMEB1*, *MECR*, *LDLRAP1*, *GPAM* and *ACSL5*. This work is a promising step toward understanding the genetic basis for chicken aldehyde traits and provides a foundation for marker-assisted selection to improve chicken flavor in the future.

## Data Availability

The data presented in the study are deposited in the Genome Sequence Archive (Wang et al., 2017) in the BIG Data Center (https://bigd.big.ac.cn/gsa/) (Members, 2019), the genomic data accession number CRA002643 and CRA002650, and the transcriptome data accession number CRA004228, CRA001908, and CRA004003.

## Supplementary material

SUPPLEMENTARY FIGURE S1 Histograms showing the distribution of relative VOC content among individuals in the GWAS.

SUPPLEMENTARY FIGURE S2 QQ-plots of VOCs from SNP GWAS.

SUPPLEMENTARY FIGURE S3 QQ-plots of VOCs from INDEL GWAS.

SUPPLEMENTARY TABLE S1 Annotated genes nearest to or harboring suggestive SNPs and INDELs for different aldehydes.

SUPPLEMENTARY TABLE S2 Main pathways of potential candidate genes.

SUPPLEMENTARY TABLE S3 Main GO terms of potential candidate genes.

SUPPLEMENTARY TABLE S4 Correlation coefficients of total aldehydes, hexanal, and nonanal content with their candidate genes expressions, respectively.
