# Supplementary figures and images for: Fatty acid metabolism-related genes are associated with flavor-presenting aldehydes in Chinese local chicken
Source: Front Genet. 2022 Aug 12;13:902180. doi: 10.3389/fgene.2022.902180 (PMC9412053; doi:10.3389/fgene.2022.902180)

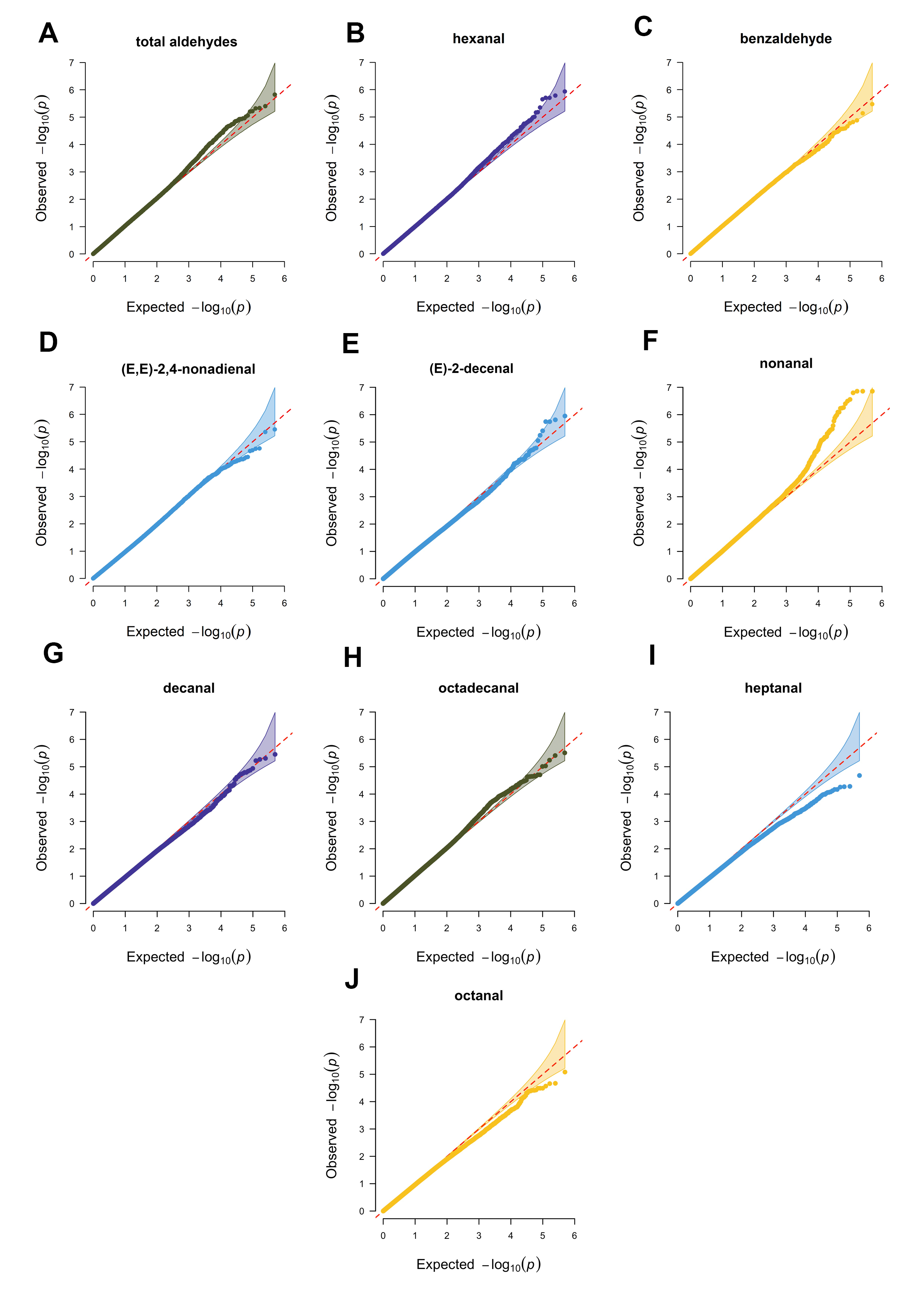

Supplement: Supplementary file 3 [file Image3.TIF]

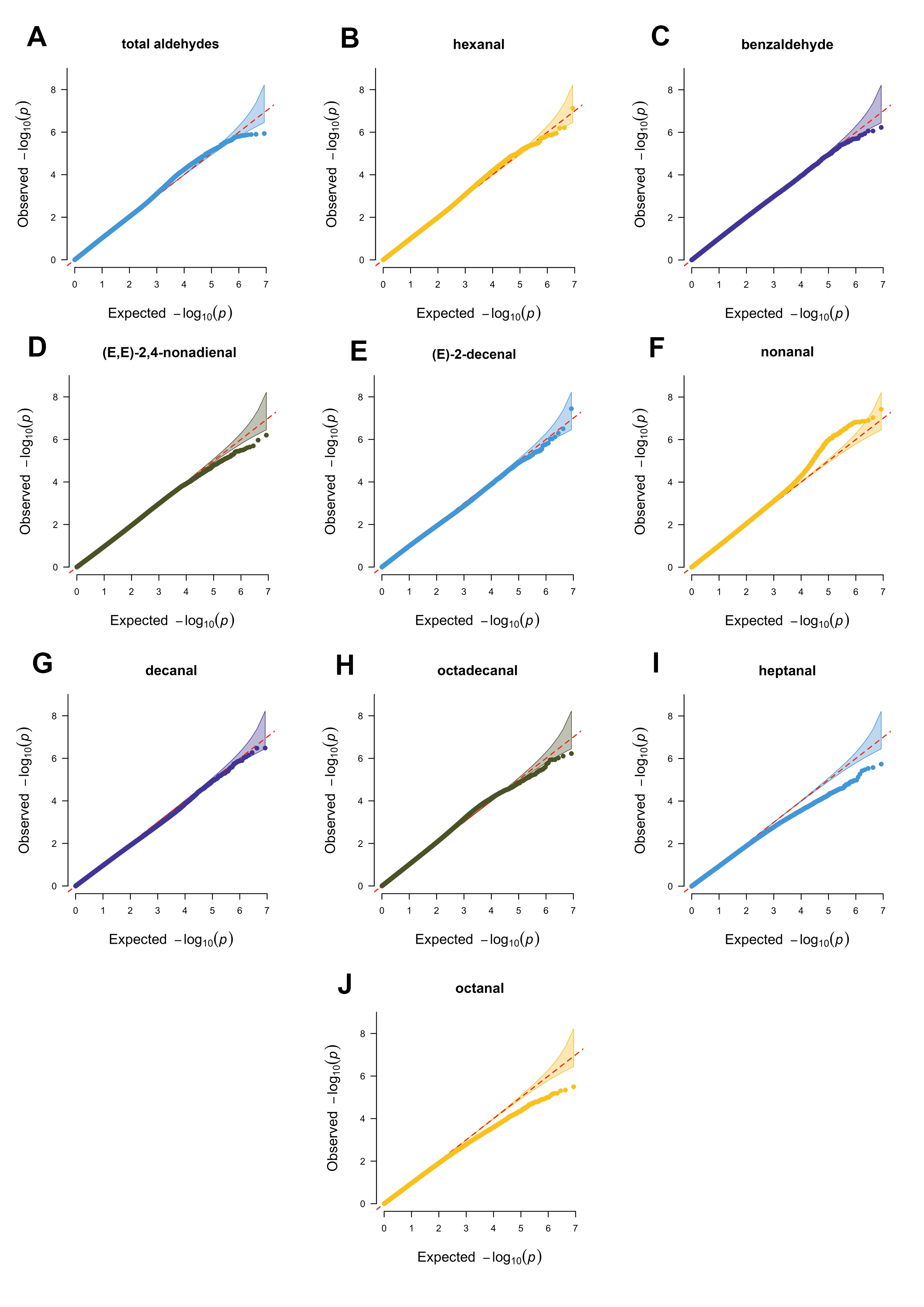

Supplement: Supplementary file 4 [file Image2.TIF]

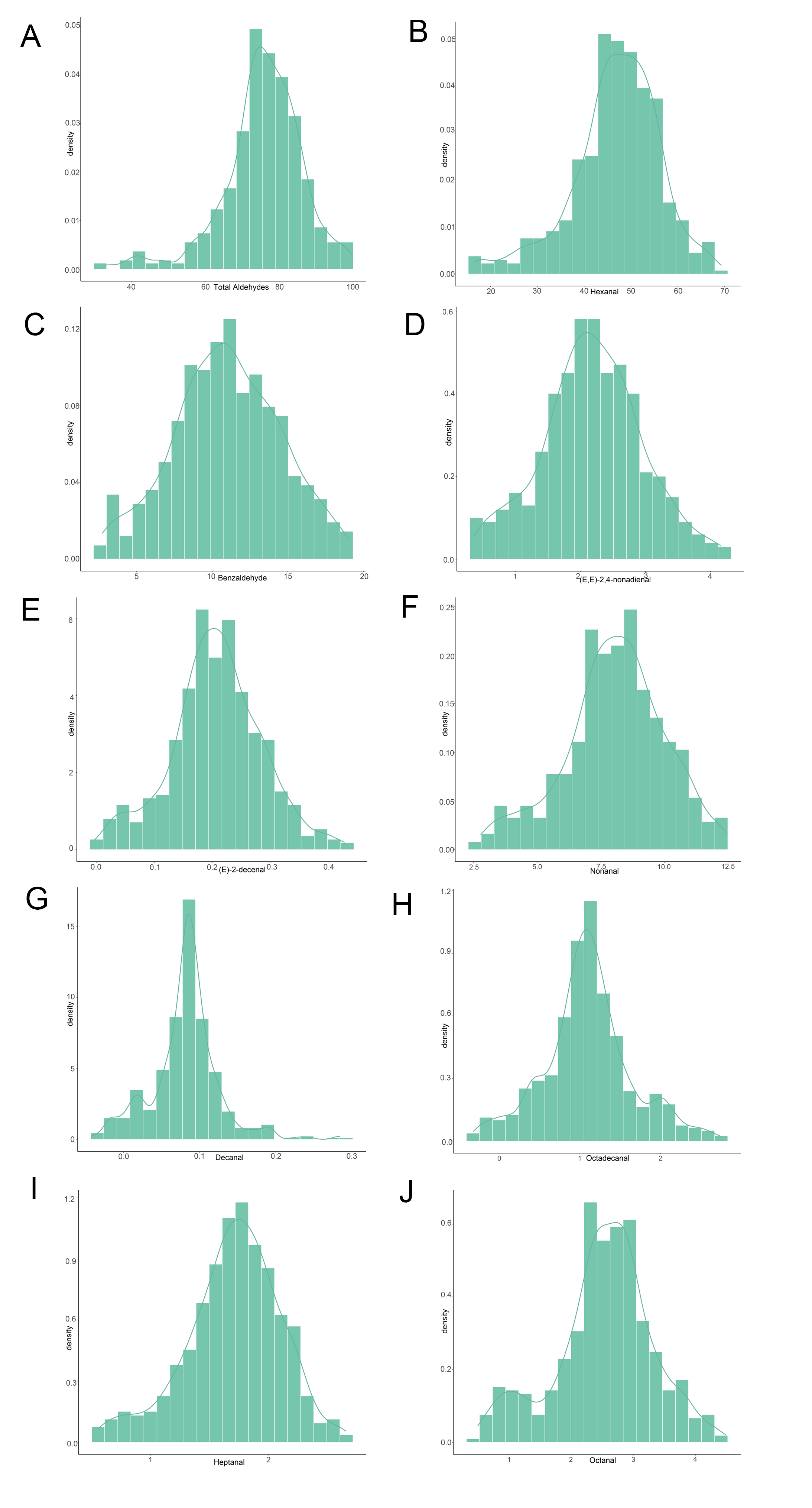

Supplement: Supplementary file 5 [file Image1.tif]
